# Supplementary material for: High-Resolution Measurements of Face-to-Face Contact Patterns in a Primary School
Source: PLoS One. 2011 Aug 16;6(8):e23176. doi: 10.1371/journal.pone.0023176 (PMC3156713; doi:10.1371/journal.pone.0023176)
Supplement: Table S1 — Comparison of the measured average numbers and durations of contacts across several studies. (DOC) [file pone.0023176.s002.doc]

| **Reference** | **Setting** | **Contact definition** | **Results** |
| --- | --- | --- | --- |
| Mikolajczyk *et al*. [4] | Survey in a primary school; 6-10 years-old children. | A person with whom the child spoke or played with in a day | 25.1 contacts per day per child  - |
| Wallinga *et al*. [7] | General population survey, divided into age classes: 1–5, 6–12, 13–19, 20–39, 40–59 and ≥60 years-old persons | Number of different conversation partners the participant encountered during a typical week by age classes. | 23.77 conversations per week (3.40 per day) held with different persons for 6-12 years-old children with other 6-12 years-old children. |
| Glass *et al*. [5] | High, middle and elementary schools surveys. Age classes: 10-12, 12-13, 14-15, 15-16, 16-17 and 17-18 years-old persons. | “An interaction with another person during which influenza could be passed. These must be within 3 feet and for a recognizable length of time” | 4.43 contacts per day for 10-12 years-old child with other 10-12 years-old children  About 1 hour per day between 10-12 years-old children |
| Zagheni *et al*. [6] | School. 5-9, 10-14 and 15-19 years-old persons. | Estimation through time-use data, under the assumption of proportionate time mixing, of the co-presence of people in the same location. | At school, 98 min (1 hour 38 min) per day between 5-9 years-old children and 113 min (1 hour 53 min) per day between 10-14 years-old children. |
| Del Valle *et al*. [21] | General population, divided into age classes: 0-4, 5-12, 13-19, 20-29, 30-39, 40-49, 50-59, 60-69 and 70-90 years old. Data are obtained from the EpiSimS agent-based simulation of an entire city, based on US census statistics. | Co-presence in the same sub-location.  The duration is deﬁned as the total length that two people spent together in the same sub-location. The durations of multiple encounters between two persons are added up and the total aggregated length gives the ﬁnal contact duration. | 3 hour 47 min between children at school (not detailed for age groups). |
| Mossong *et al*. [8] | General population in 8 European countries, divided into age-classes: 0-4, 5-9, 10-14, 15-19, 20-29, 30-39, 40-49, 50-59, 60-69 and ≥70 years-old persons | Either skin-to-skin contact such as a kiss or handshake (a physical contact), or a two-way conversation with three or more words in the physical presence of another person but no skin-to-skin contact (a nonphysical contact) | Average number on all reported contact persons (physical and non-physical contacts) per day per person:  From 2.25 to 11.88 between 5-9 years old children, depending on the country  From 3.58 to 14.56 between 10-14 years-old children depending on the country |
| Salathé *et al*. [3] | US high school: students, teachers and staff. | Electronic devices (motes). A close proximity record CPR represents one close (≤3 meters) proximity detection event between two electronic devices. A contact is defined as a continuous sequence of CPR between two motes. | On average 1900 contacts per student per day, lasting on average about 1 minute. Broad distributions of the duration of contacts and of the cumulative time spent in proximity by two individuals. Each individual has contact with an average of 300 distinct other individuals. |
| Stehle *et al*. (present study) | Primary school: 6-12 years-old children | RFID devices that exchange radio packets only when the individuals wearing them face each other at close range (about 1 to 1.5 m) | On average 323 contacts per child per day, lasting on average 33 seconds, with on average 47 other distinct individuals. Cumulated contact time of each individual of 176 min (2 hours 56 min) per day on average. Broad distributions of the duration of contacts and of the cumulative time spent in proximity by two individuals. |
